# Dr. Kentish's Answer to Dr. Wood's Charge of Literary Piracy; and of His Unprincipled Suppression of the Source from Whence He Drew His Information

**Published:** 1808-05

**Authors:** E. Kentish

**Affiliations:** Bristol


### Dr. Kent.sh's *Answer to Dr.* Wood's *Charge of Literary Piracy; and of his unprincipled Suppression of the Source from whence he drew his Information.*

To enable you the more clearly to comprehend my statement, I shall begin by laying before you Dr. Wood's charges; he says, "In 1793, I collected a few ideas on the subject, which, together with some general principles, I published in that year. To exemplify one of the said general principles, I used the following words," "we can apply not only with safety, but with the best effect, ardent spirits to a part burned or scalded." "Were it necessary, I could produce such circumstantial evidence, as would be admitted even in a Court of Justice, that the above words were the origin of the stimulating plan of cure by Dr. Kentish in this place, and of his consequent publication on that subject. When that publication appeared, I knew that some of the first surgeons here pointed out my hint as the first they had received on the subject. It was never even alluded to by Dr. Kentish, though I then, and ever since, have openly maintained its priority. At the time I published those general principles, the emollient plan of cure was, I have always understood, universally used; cold applications were not, I believe, even thought of."—"It is said, that an irregular practitioner in this neighbourhood has long used spirituous applications in cases of burns; but at the time I exemplified my general principle, by the above instance of the efficacy of ardent spirits to parts burned, or scalded, I had resided a very short time in this place, and was equally ignorant of the practice of the regular and irregular practitioner. A common desire for justice alone has induced me to make this public statement, which I doubt not I can maintain. Yet I by no means consider the value of the discovery, (if it may be so called) of such importance as to be worth a competition." Thus stands Dr. Wood's denunciation of me to the whole of my brethren of the faculty; and this he does for common justice. It is a strange love of justice that has seized the Doctor! It is now eleven years since I published my first Essay on Burns, five of which I passed in the same town with him, when once a month we met in a Medical Society of about twelve members, at each other's houses, to converse on medical subjects, and to circulate medical books; will it be believed when asserted, that during this period of five years, Dr. Wood never spoke to me upon the subject, nor did he ever, in my presence, make any such claim to the society; what is it then that has awakened the Doctor's sleeping justice? But let us examine this prolific idea from whence all mine sprung; "we can apply not only with safety, but with the best effect, ardent spirits to a part burned or scalded." Does this convey a more precise idea than what I had from Heister? (vide 1st Essay, page 14.) "Among gentle astringents, common spirits, or rectified, or even camphorated spirit of wine, may be reckoned, provided the injured parts be dipped into it, or be carefully bathed with rags moistened in the spirit." Does not Heister give me as much information on the subject as Dr. Wood; but, further, page 27, 1st Essay—"But spirit of wine, we are assured by Sydenham, is preferable to any other remedy as yet known; namely, if linen rags are dipped, and applied to the burnt parts, and the application of them repeated till the pain excited by the fire is entirely removed; and afterwards the same application he would have repeated only twice a day." Sydenham, above a century before, was certainly as minute in the manner, as he was decided in the advantage to be derived from the application of spirit in burns, as Dr. Wood was in 1793; but had the ideas of Heister, or of Sydenham, produced any plan or system which philosophy could regard as dictated by common sense, until the period when I wrote my Essay? I should be glad to know upon what principle Dr. Wood supposes his idea should produce so much, when the ideas of at least as great men as himself, should have produced no effect at all? I here confess to Dr. Wood, that had I seen his work previous to the publishing of mine, I should have ranked him among those moderns who recommend spirit; I suppose I may now add among those who recommend cold water: but I here solemnly assert, that I never saw, read, or heard of the passage alluded to by Dr. Wood until after I had published my first Essay; and this I do on the word of honour of a gentleman. But happily for me, my justification does not depend upon this assertion only; no, the Doctor has fortunately supplied me with dates, which will put the matter beyond the possibility of a doubt. Dr. Wood says, "in 1793, I collected a few ideas on the subject, which, together with some general principles, I published in that year," namely, the year 1793; now comes a stubborn fact, which at once does away Dr. Wood's accusation, vide page 148, 1st Essay.

"CASE.—G. H. a pitman, aged about 34, belonging to a coal a coal work in the neighbourhood, in the month of November, 1792." It is unnecessary to repeat the whole of the case, but it must be observed that this case is the first recited case in which the whole of the third, or what I termed the complete method of treatment is delivered, viz. Perfect external and internal stimulation on the happening of the accident; then gradually diminishing their use, until we arrive at the period when the least possible stimulation, or rather the most soothing plan, until health be restored. Though this is the first case recorded, yet it is not by several the first so treated; the first idea I got of using stimulants was in Paris, in the year 1787, and the year 1788, in attending Fourcrois's Lectures, at the Lyceum; he mentioned the practice of Mons. Le Sage, who recommended the use of the fluor volatile alcali. Fourcroy supposed it might combine with some of the disengaged matter of heat, and carry it off before it could combine with the living fibre, vide Essay 1, p. 125. On my return to Newcastle, at the latter end of 1788, I had determined, in my own mind, on the use of this remedy; in 1789 and 1790, I used it with beneficial effect; looking upon it as a stimulant I tried some others; and in the year 1792, I had so far completed my practice as to give a detail of a severe case, treated according to the principles I have laid down in my first, and more fully exemplified by the testimonies of some of the ablest surgeons in the kingdom, in a second Essay.

Now, Gentlemen, upon what principle Dr. Wood is to substantiate before a Court of Justice, that my practice in 1792 was influenced by a publication of his in 1793, I am perfectly at a loss to divine; but as he has made the assertion, the task must be left to him. After his accusation, he finishes with a degree of nonchalance, saying, "Yet I by no means consider the value of the discovery to be of such importance as to be worth a competition." Now, in this also, I beg leave to differ with Dr. Wood, when I review the labyrinth of opinions on this subject, from the first recorded Medical histories to the time I wrote my book; that there was nothing to guide the choice of the inquirer; that there was no principle to lead the practitioner; every thing had been tried, nothing new could be thought of, or recommended; but still every thing was full of doubt. If then, in this state, I was fortunate enough to find a principle which might guide the practitioner to give relief to his patient in all stages and degrees of this accident, even from the slightest scorched finger of the most delicate female, to the total immersion of the frame of a Hercules in liquid fire; if then I have done this; and I have undoubted testimonies from some of the ablest surgeons in the kingdom that I have done so, I do not hesitate to say, that I think myself entitled to some degree of merit, and that I shall contend the point with Dr. Wood; for I have not confined my practice *merely* to the application of stimulants externally, but have, contrary to the practice, advice, and principles of every known author, recommended the strongest of all internal stimulants, as placing the system in the best possible attitude of defence against the tremendous shock it sustained; and for this I have the decided opinion of its beneficial effects, of the most capable to form such an estimate, viz. some of the most eminent surgeons who have tried it. These principles, on my part, have not been established without personal danger, much difficulty, and considerable expence. The irregular practitioner Dr. Wood alludes to, was called in to a terrible accident at a colliery where I attended; thirteen or fourteen men were severely burnt, one died in twenty-four hours, several others were in extreme danger; the sovereign mob had a variety of opinions; some wished me to continue; others would have the irregular alluded to. They made their election, and we both attended those who wished it, not together, but separately; such of the people as wished for a change were allowed to do so. As well as I can recollect, three or four chose the *new* man; one of them died a few days after he had attended him. In an alehouse in the adjoining village, this irregular, surrounded by the colliers, was expatiating upon the death of the man, and had the temerity to say that I had killed him, for if he had seen him at first he could have saved him.

Those colliers, who, over a pot of ale, wished to prove themselves logicians, as they naturally are all politicians, said, "why, if he killed my friend, or brother Jack, there can be no harm in killing him." Now it fortunately happened for me, that the landlord of the Inn was a sensible intelligent man, who had been butler in a family I attended, and who himself was a patient of mine; he immediately saw the dangerous tendency of their reasoning, and that my life was in hazard from the perverted judgment of these deluded men, he therefore informed me of the danger I was in.

To convince these men that I was under the protection of the law, I prosecuted Mr. Huntley (that is the name of of the irregular alluded to), and laid my damages at a thousand pounds. The facts were so evident, that his counsel advised him not to allow it, if possible, to go into court. Upon submitting to make a most humiliating apology, and asking pardon in the public papers, I consented to withdraw the suit. Thus terminated my struggle with this (I dare not allow my pen to name him) which ended in my own preservation, and the quiet of my Medical Brethren in Newcastle.

Among the variety of remedies, it was not without difficulty, and even it is seen danger, that I could apply any great change upon such materials as I had to work upon. I was ready to hear and learn from the nostrums of all the old women of the country. I had a very intelligent pupil (Mr. Bell), who had some idea that cold was a proper application; he tried it upon his own person, but the result was so little satisfactory that he became afterwards a proselyte, and one of my firmest advocates for the *whole* of my principles, as may be seen in my Second Essay; and in several cases he has sent to the Medical Journal. Probably these are difficulties which can only be appreciated by those who have had nearly similar ones to overcome. An extensive correspondence, in consequence of my publications, with the faculty in the most distant parts of the kingdom, much more than absorbed the small profits arising from my publication, though the whole were sold, and are now out of print.

Thus, I think, I have made out my assertion, that I have, in establishing these principles, had to contend with personal danger, with embarrassing difficulties, and with considerable expence.

Previous to the publication of my first Essay, I had the satisfaction of knowing that by the alteration of practice I had preserved many valuable lives to their families, which by former modes would have been lost. I dedicated my Essay with some confidence to the "Proprietors of Collieries upon the River Tyne," looking upon them as the natural guardians of their workmen, and acutely alive to every thing that interested their welfare; one of them very handsomely proposed making me a present of a piece of plate for my services, but some others objected, as the trade at that time was not very flourishing.

As the Medical Gentlemen of the neighbourhood have now had more time to appreciate the value of my publication, and have sanctioned the principles, as producing the same good in their hands as they did in mine; I hope, when when they have "Success in the coal trade," they will recollect they are still my debtor. It would lead me into too wide a field were I, at present, to enter upon the comparison of Dr. Kinglake's doctrine and my own. I shall only add, that I have not been a bigot to my own system; that I wrote to the Newcastle Medical Society, wishing them duly to appreciate the facts brought forward by the opposite party, and offering some slight modifications, by which I trusted benefit in the treatment might ensue, for from that source alone am I capable of pursuing the subject, as I am not now personally so situated, as to be likely to determine the comparative value of the two modes myself. My friend, Dr. Ramsay, writes to me as follows: "I took the first opportunity of submitting your modified doctrine of burns to the society. In the practical use of the old Kentishian theory, the slight variation suggested was very well received; *but every one*, especially Horn, conceived that any compromise with the cold water disciples ought to be resisted, as the *uniform success of the improved treatment had set the subject at rest in their minds*. One of Mr. Bulman's children, of Coxlodge, was lately dreadfully burnt, by her clothes taking fire, and under Mr. Horn's care, and *your* printed prescriptions, the recovery has been complete."

Having, I trust, satisfactorily refuted Dr. Wood's charge, I cannot leave my principles in the hands of better defenders than those Gentlemen, who enjoy the field of practice in which I acquired them, and who acknowledge that my delineation of nature, in this department, is just, and has "set the subject at rest in their minds."

E. KENTISH, M. D.

*Bristol, March* 29, 1808.

